# **Sickness absence among blue-collar workers in the retail and wholesale industry during the COVID-19 pandemic; a longitudinal cohort study**

**DOI:** 10.1038/s41598-025-97025-z

**Published:** 2025-04-20

**Authors:** Kristin Farrants, Lukasz Cybulski, Kristina Alexanderson

**Affiliations:** Division of Insurance Medicine, Department of Clinical Neuroscience, Karolinska Institutet, Stockholm, 171 77 Sweden

**Keywords:** Risk factors, Respiratory tract diseases, Occupational health, Epidemiology

## Abstract

Sickness absence (SA) changed in various occupations during the COVID-19 pandemic. The aim was to investigate the prevalence of all-cause sickness absence (SA) during the COVID-19 pandemic in relation to in the preceding years, as well as factors associated with all-cause SA and SA due to COVID-19 and COVID-like diagnoses during the COVID-19 pandemic among blue-collar workers in the retail and wholesale industry. A population-based longitudinal cohort study using microdata linked from nationwide registers in Sweden. All 297 378 blue-collar employees aged 18–67 years in wholesale and retail in 2019 were followed during 2016–2021 regarding SA in spells > 14 days. Yearly prevalence rates were calculated for all-cause SA in sociodemographic and occupational groups. Logistic regression was used to calculate odds ratios (OR) and 95% confidence intervals (CI) for all-cause SA and SA due to COVID-19 or COVID-like diagnoses in 2020 and 2021. The annual prevalences of SA were 7.5-8% in 2016–2018, 10% in 2020, and 9% in 2021. The prevalence of SA due to COVID-19 or COVID-like diagnoses was 2.1% in 2020 and 1.6% in 2021. The OR was higher in the older age groups (OR age 55–64 = 2.38, 95% CI 2.20–2.57 compared to age 25–34). There were few significant occupational differences, however, warehouse and terminal staff had a higher OR (1.37, 1.27–1.48) than sales assistants, daily goods. While SA rates increased during the COVID-19 pandemic, the distribution of SA between sociodemographic or occupational groups did not change markedly. The distribution of SA due to COVID-19 and COVID-like diagnoses was similar to all-cause SA.

## Background

The COVID-19 pandemic affected many areas in societies, including the labour market, public health, and sickness absence (SA)^1–3^. In Sweden, SA increased greatly during the first half of 2020 compared to prior years^4,5^, especially due to what the Social Insurance Agency calls “COVID-related” diagnoses, but also due to mental and musculoskeletal diagnoses^4^. For instance, there were twice as many new SA spells > 14 days in April 2020 than in April 2019^5^. Most people with COVID-19 did not require SA > 14 days due to that diagnosis because they were able to either manage their work tasks with the infection by working from home or because they returned to work within 14 days. The increase in SA due to these diagnoses therefore represented more severe and long-lasting cases^6^.

While there was an increase in SA due to COVID-like diagnoses and due COVID-19 itself, the patterning of SA during the pandemic might have been influenced by other diagnoses as well, both due to individuals’ changed behaviour in response to the pandemic, as well as due to changed regulations governing SA, which were applied to all diagnoses, not just to COVID-19 or COVID-like diagnoses^5^. Furthermore, many people in paid work changed to working from home, in line with the governmental recommendations intended to limit disease transference^7,8^, which may have impacted their need for SA when they had various diseases or injuries due to, e.g., possibilities to rest during the working day or not having to commute^8^. However, it is not possible to work remotely in all types of occupations, especially not in many of the occupations usually classified as blue-collar work^9^ and so-called essential work^10^, some of which are in the retail and wholesale sector (such as sales of daily goods, logistics and distribution)^10^.

A great deal of the research on SA during the COVID-19 pandemic has been on healthcare staff^11–13^, many of whom were highly exposed to people with COVID-19 infections, e.g., patients, colleagues, as well as other passengers when commuting to work on public transportation. However, retail and wholesale workers might also have been impacted by the COVID-19 pandemic. The recommendations about keeping distance and avoiding crowds might have led to people avoiding physical shops, which has sometimes led to furlough, cut-downs, or closures, but also an increase of e-trade and the occupations associated with that (e.g., warehouse staff, packing, logistics, etc.)^14^. Most of the occupations in this sector, both in physical shops and in the e-trade sector, require the worker to be physically present, and can thus expose the worker to infection, both at work, and potentially when commuting to and from work if it is done by public transport.

Approximately 10% of all in work or studies in Sweden, work in the retail and wholesale industry. Of those who are aged 16–24 and in work or studies, the proportion is even higher: 18% of them work in retail and wholesale^15^. Many work part-time: approximately 70% of employees in retail (75% of women, 60% of men)^16^. The mean salary for fulltime workers in the sector is approximately 75% of the mean salary in Sweden for fulltime workers, however, due to the high prevalence of part-time work, the actual salary is much lower^17,18^. Despite the sector being a large part of the Swedish labour market, it has received relatively little attention from the scientific community.

Even before the pandemic, little was known about SA among blue-collar workers in the retail and wholesale industry. Of the few studies conducted, some have shown that wholesale and retail workers can be exposed to the adverse working conditions that can be risk factors for SA, such as repetitive movements^19–21^, adverse psychosocial working environment^19^, and shift work^22^. Also, some studies, particularly on cashiers, have shown relatively high rates of musculoskeletal disorders^19–21^. However, we have only found one study regarding SA in this sector, namely one of retail workers in Finland; it showed that shift work and long working hours were associated with SA^22^. There have, to the best of our knowledge, been no studies comparing SA in specific occupations within retail and wholesale at a detailed level. Previous studies on the general population have found differences by sociodemographic and occupational factors^23,24^, however, no such studies exist on blue-collar workers in retail and wholesale.

*The aim* was to investigate the prevalence of all-cause sickness absence (SA) during the COVID-19 pandemic in relation to in the preceding years, as well as factors associated with all-cause SA and SA due to COVID-19 and COVID-like diagnoses during the COVID-19 pandemic among blue-collar workers in the retail and wholesale industry.

## Methods

This is a population-based longitudinal cohort study of SA among privately employed blue-collar workers in the retail and wholesale industry in Sweden.

### Data and study population

We used anonymised data from three nation-wide Swedish administrative registers, linked at the individual level through a unique ten-digit identifier assigned to all residents in Sweden^25^. Data were accessed for research purposes from February 2022-August 2023, and we included the years 2016–2021. To delineate the study cohort and to obtain annual information on sociodemographic and work-related factors (age, sex, country of birth, type of living area, family situation, educational level, income, occupational code, sector, emigration, and branch of industry) between the years 2016 through 2020 we utilised the Longitudinal Integration Database for Health Insurance and Labour Market Studies (LISA) held by Statistics Sweden. We used the MicroData for Analysis of the Social Insurance database (MiDAS) held by the Swedish Social Insurance Agency to acquire information on SA spells lasting 15 days or longer, including data on dates, extent (i.e., part- or full-time), primary diagnosis, employment status at the start of SA spell and disability pension (dates and extent) in the period 2016–2021. Finally, we obtained information regarding date of death from the Cause of Death Register held by the National Board of Health and Welfare.

The study population included all individuals aged 18–67 years who were registered as living in Sweden on both 31 December 2018 and 31 December 2019 and who had an occupational code that indicated a blue-collar occupation according to the Swedish Standard for Occupational Classification (SSYK, the Swedish version of the International Classification of Occupations, ISCO). We also required individuals to be employed at a private sector company in 2019 in the retail and wholesale industry as defined by the Swedish Standard Industrial Classification (SNI), and to have had income from work, parental benefits, and/or SA/DP that amounted to at least 8370 SEK (approx. 790 EUR by the 2019 exchange rate). This income limit corresponds to 75% of the necessary income to qualify for SA benefits from the Social Insurance Agency, since without this adjustment, the income of people with both low incomes and SA might have been too low for them to be included in the study, given that in many cases, only 75% of the work income is covered by SA benefits^26^. Those who were employed in the public sector, self-employed, or who had full-time disability pension all of 2019 were excluded, as were those who died or emigrated before 1 January 2020. The final study cohort included 308 480 individuals in 2019.

For analyses on SA for each of the seven years, we each year only included those who were aged 18–67, had an income from paid work over 75% of the minimum income to qualify for SA benefits, and did not have ongoing full-time disability pension the year before each respective year. However, due to delays in data delivery, income data was not available for 2021. Those whose income was below the threshold to qualify for SA benefits in 2020 were, therefore, also excluded in 2021. Individuals who died or emigrated were excluded from the year after their death or emigration. Individuals who did not live in Sweden in 2016 or 2017 were excluded from the year(s) they did not live in Sweden. For the logistic regression analyses regarding all-cause SA and regarding SA due to COVID-19 or COVID-like diagnoses, we excluded those who in 2020 were aged above 67 and had an income of less than 75% of the minimum income to qualify for SA benefits, which in 2020 was 8514 SEK (approx. 810 EUR by the 2020 exchange rate).

### Public sickness absence insurance in Sweden

The public sickness absence insurance in Sweden has been described in detail previously^26,27^. In brief, the sickness absence insurance covers all individuals with an income from work or unemployment benefits in case of reduced work capacity due to morbidity. For those who are employed, the employer provides sick-pay from day 2 to day 14, after a first waiting day, and the Social Insurance Agency pays SA benefits from day 15 (however, unemployed individuals get their benefits from the Social Insurance Agency after the waiting day, and self-employed can have more waiting days). A physician certificate is required after seven days of self-certification, although during some of the COVID-19 pandemic this was temporarily changed to 21 days.

All residents in Sweden aged 19–64 years are eligible for disability pension if their work capacity is long-term or permanently reduced due to morbidity.

Both SA and disability pension can be granted for 25, 50, 75, or 100% of ordinary work hours. SA benefits cover 80% and disability pension benefits 64% of lost income, both up to a certain level.

### Variables

We used information on the following variables from 2019 unless otherwise noted: *Sex*: woman or man; *Age*: 18–24, 25–34, 35–44, 45–54, 55–64, or 65–67 years; *Country of birth*: Sweden, other Nordic country, other EU-27, or rest of world including missing (*N* = 26, < 0.1%); *Educational level*: elementary school (≤ 9 years or missing (*N* = 2259, 0.8%)), high school (10–12 years), or college/university (≥ 13 years); *Family situation*: married/cohabiting with children < 18 years at home, married cohabiting without children at home, single with children at home, or single without children at home; *Type of living area* based on the Degree of urbanisation classification^28^: large city (Stockholm, Gothenburg, or Malmö), medium-sized town, or small town/rural; *Income*: categorised according to the price basic amount (a yearly amount set for indexing purposes) into 8514-92 999 SEK, 93 000-185 999 SEK, 186 000-348 749 SEK, 348 750 − 464 999 SEK, ≥ 465 000 SEK; *Occupation*: categorised into the following 15 groups using SSYK: warehouse and terminal staff, sales assistant for daily goods, sales assistant for specialist trade, cashiers, motor vehicle mechanics and repair personnel, construction workers, craft workers, machine and process operators, mechanics, technicians, repair, installation etc., transport occupations, security staff, porters, cleaners, etc., other logistics, other service, other sales, or other.

*Sickness absence*: In this study, data on SA with benefits from the Social Insurance Agency were used. SA spells < 15 days were not included in the study, so as not to introduce bias regarding those who might have been unemployed for part of the year.

*Sickness absence due to COVID-19 or to COVID-like diagnoses*: SA spells due to ICD-10 diagnoses U07, U09, or U10 were categorised as COVID-19 diagnoses^29^. SA spells due to certain diseases of the respiratory system diagnoses (J00, J02, J04, J06, J11, J12, J16, J18, J20, J21, J22, J44, J45, J46, J80, J96, J98), certain infectious and parasitic diseases (A08, A09, B09, B34, B97, B99), and the following symptoms, signs and abnormal clinical and laboratory findings, not elsewhere classified (R00, R05, R06, R07, R20, R21, R23, R43, R50, R51, R53, R65) were categorised as COVID-like diagnoses, following the Social Insurance Agency’s practice (they call it “COVID-related” SA)^5,30^.

### Statistical analyses

We first estimated the number and proportion of cohort members with SA due to any diagnosis in 2016, 2017, 2018, 2019, 2020, or 2021, respectively, to determine if the rates or distribution of SA were different in 2020 from the preceding years. We then estimated the numbers and proportions of individuals in the cohort who had SA due to a COVID-19 or COVID-like diagnosis in 2020 and 2021, respectively. We used logistic regression to calculate the crude and adjusted odds ratios (OR) and 95% confidence intervals (CI) for having had SA due to COVID-19 or COVID-like diagnoses as well as all-cause SA in 2020 and 2021. Adjusted models were mutually adjusted for age, sex, educational level, family situation, birth country, type of living area, income, and occupation. The variables were chosen based on having associations with SA in previous literature, and were mutually adjusted since they are also related to each other^23,24,26,31^.

## Results

Table 1 shows the distribution of sociodemographic factors in the cohort of the 297 378 blue-collar workers in the retail and wholesale industry in 2019. There were about the same proportions of women and men, and more people who were < 25 (26.5%) than > 55 (11.7%) years old. The majority of the cohort was born in Sweden (84.5%), had attained at least some high school education (67.3%), and were single without children living at home (60.2%). In terms of occupation, the three largest groups were sales assistant for specialist goods (31.4%), sales assistant for daily goods (26.1%), and warehouse and terminal staff (11.6%).

**Table 1:** Sociodemographic characteristics of the cohort of 297 378 blue-collar workers in the retail and wholesale industry in 2019.

	All
	*N* = 297,378 (100%)
Sex	
Women	142,189 (47.8%)
Men	155,189 (52.2%)
Age in years	
18–24	78,289 (26.3%)
25–34	91,523 (30.8%)
35–44	49,597 (16.7%)
45–54	44,571 (15.0%)
55–64	30,536 (10.3%)
65–67	2862 (1.0%)
Educational level	
College/university	54,424 (18.3%)
High school	201,609 (67.8%)
Elementary	41,345 (13.9%)
Family situation	
Single without children at home	179,107 (60.2%)
Single with children at home	10,332 (3.5%)
Married/cohabiting without children at home	35,209 (11.8%)
Married/cohabiting with children at home	72,730 (24.5%)
Birth country	
Sweden	251,767 (84.7%)
Other Nordic country	3129 (1.1%)
Other EU-27	7348 (2.5%)
Rest of the world	35,134 (11.8%)
Type of living area	
Large city	115,791 (38.9%)
Medium-sized town	127,086 (42.7%)
Small town or rural	54,501 (18.3%)
Occupation	
Sales assistant for daily goods	77,966 (26.2%)
Sales assistant for specialist trade	93,479 (31.4%)
Cashiers	5979 (2.0%)
Warehouse and terminal staff	34,674 (11.7%)
Motor vehicle mechanics and repair personnel	22,220 (7.5%)
Construction workers	6195 (2.1%)
Craft workers	3156 (1.1%)
Machine and process operators	3523 (1.2%)
Mechanics, technicians, repair, installation etc.	11,464 (3.9%)
Transport occupations	4567 (1.5%)
Security staff, porters, cleaners, etc.	6667 (2.2%)
Other logistics	6032 (2.0%)
Other service staff	12,753 (4.3%)
Other sales staff	6104 (2.1%)
Other occupation	2599 (0.9%)
Income from work in 2019	
8514-92,999 SEK	25,215 (8.5%)
93,000–185,999 SEK	43,657 (14.7%)
186,000–348,749 SEK	136,792 (46.0%)
348,750–464,999 SEK	72,988 (24.5%)
≥ 465,000 SEK	18,726 (6.3%)

Table 2 shows the number and proportion (%) of individuals who had any SA due to any diagnosis in 2016, 2017, 2018, 2019, 2020, or 2021, respectively. These proportions were fairly similar in the four years 2016–2019, around 8%, and increased by 2% units (corresponding to a 25% increase) to 10% in 2020, but decreased slightly to 9% in 2021. The rates of people with SA in different socioeconomic groups did not vary much over the years. However, SA rates increased in all groups in 2020 and 2021 relative to previous years, and this was particularly noticeable among those born outside Sweden.

**Table 2:** Number of people with sickness absence (SA) irrespective of diagnosis, each of the years 2016–2019 among the cohort of blue-collar workers in the retail and wholesale industry in 2019 among those who were aged 18–67 and in paid work each respective year, and the percentage they constitute of all workers in each respective category.

Total with any SA	2016 (*N* = 258,457)	2017 (*N* = 277,135)	2018 (*N* = 294,806)	2019 (*N* = 308,480)	2020 (*N* = 296,754)	2021 (*N* = 295,766)
	*n* = 21,224 (8.21%)	*n* = 21,361 (7.71%)	*n* = 22,763 (7.72%)	*n* = 23,935 (7.76%)	*n* = 30,359 (10.2%)	*n* = 26,690 (9.02%)
Sex						
Women	12,204 (10.0%)	12,111 (9.2%)	13,005 (9.3%)	13,435 (9.1%)	16,340 (11.5%)	14,464 (10.2%)
Men	9020 (6.6%)	9250 (6.3%)	9758 (6.3%)	10,500 (6.5%)	14,019 (9.1%)	12,226 (7.9%)
Age						
18–24 years	1058 (2.3%)	1710 (2.9%)	2374 (3.3%)	3033 (3.7%)	4169 (5.3%)	3853 (4.9%)
25–34 years	6571 (7.6%)	6643 (7.4%)	7084 (7.7%)	7550 (8.0%)	9146 (10.0%)	7902 (8.6%)
35–44 years	4516 (9.5%)	4363 (9.0%)	4434 (8.9%)	4612 (9.1%)	5958 (12.0%)	5115 (10.3%)
45–54 years	4673 (10.7%)	4387 (9.9%)	4537 (10.1%)	4558 (10.0%)	6147 (13.8%)	5589 (12.5%)
55–64 years	3920 (12.5%)	3856 (12.2%)	3958 (12.5%)	3954 (12.3%)	4777 (15.6%)	4181 (13.7%)
65–67 years	486 (12.8%)	402 (10.6%)	376 (9.9%)	228 (5.9%)	162 (7.3%)	50 (3.9%)
Educational level						
College/university	2915 (5.7%)	2874 (5.4%)	2962 (5.4%)	3099 (5.4%)	4306 (7.9%)	3752 (6.9%)
High school	14,679 (8.4%)	14,858 (7.9%)	15,831 (7.8%)	16,642 (8.0%)	20,759 (10.3%)	18,383 (9.2%)
Elementary	3630 (11.0%)	3629 (10.4%)	3970 (10.6%)	4194 (9.6%)	5294 (12.9%)	4555 (11.2%)
Family situation						
Single without children at home	8987 (6.3%)	9514 (6.0%)	10,427 (6.0%)	11,504 (6.2%)	15,345 (8.6%)	13,405 (7.5%)
Single with children at home	1437 (14.3%)	1335 (13.1%)	1339 (12.8%)	1446 (13.6%)	1643 (15.9%)	1542 (14.9%)
Married/cohabiting without children at home	3987 (11.2%)	3840 (10.6%)	3960 (10.9%)	3874 (10.5%)	4988 (14.3%)	4391 (12.8%)
Married/cohabiting with children at home	6813 (9.7%)	6672 (9.3%)	7037 (9.7%)	7111 (9.6%)	8383 (11.5%)	7352 (10.1%)
Birth country						
Sweden	17,923 (8.1%)	18,063 (7.6%)	19,119 (7.6%)	19,789 (7.6%)	24,373 (9.7%)	21,712 (8.7%)
Other Nordic country	312 (11.1%)	279 (9.3%)	316 (10.0%)	335 (10.1%)	445 (14.3%)	337 (10.9%)
Other EU-27	515 (8.4%)	551 (8.3%)	589 (8.1%)	670 (8.6%)	842 (11.5%)	739 (10.1%)
Rest of the world	2474 (8.9%)	2468 (8.0%)	2739 (8.0%)	3141 (8.5%)	4699 (13.4%)	3902 (11.1%)
Type of living area						
Large city	7474 (7.2%)	7614 (7.0%)	8213 (7.1%)	8700 (7.2%)	11,872 (10.3%)	9967 (8.6%)
Medium-sized town	9306 (8.2%)	9384 (8.0%)	9905 (7.9%)	10,395 (7.9%)	12,878 (10.2%)	11,674 (9.2%)
Small town or rural	4448 (8.9%)	4363 (8.5%)	4645 (8.6%)	4840 (8.6%)	5609 (10.3%)	5049 (9.3%)
Occupation						
Sales assistant for daily goods	5609 (8.8%)	5456 (7.8%)	5899 (7.8%)	6268 (7.8%)	7926 (10.2%)	6912 (8.9%)
Sales assistant for specialist trade	6349 (7.7%)	6381 (7.2%)	6768 (7.3%)	6833 (7.0%)	8780 (9.4%)	7770 (8.4%)
Cashiers	411 (8.8%)	433 (8.2%)	463 (8.0%)	443 (7.1%)	597 (10.0%)	525 (8.8%)
Warehouse and terminal staff	2895 (9.3%)	3067 (9.3%)	3243 (9.3%)	3618 (10.1%)	4726 (13.6%)	4087 (11.8%)
Motor vehicle mechanics and repair personnel	1500 (7.3%)	1517 (7.1%)	1615 (7.3%)	1784 (7.8%)	2244 (10.1%)	1993 (9.0%)
Construction workers	447 (7.8%)	456 (7.6%)	496 (8.0%)	543 (8.5%)	640 (10.4%)	601 (9.8%)
Craft workers	265 (9.0%)	268 (8.7%)	252 (7.9%)	283 (8.6%)	337 (10.7%)	286 (9.1%)
Machine and process operators	289 (8.8%)	261 (7.6%)	253 (7.1%)	280 (7.6%)	332 (9.4%)	303 (8.7%)
Mechanics, technicians, repair, installation etc.	807 (7.3%)	837 (7.4%)	888 (7.6%)	911 (7.7%)	1086 (9.5%)	956 (8.4%)
Transport occupations	377 (9.0%)	370 (8.4%)	401 (8.7%)	407 (8.6%)	539 (11.9%)	454 (10.1%)
Security staff, porters, cleaners, etc.	529 (9.1%)	529 (8.4%)	530 (7.9%)	549 (7.7%)	659 (9.9%)	619 (9.4%)
Other logistics	407 (7.4%)	416 (7.2%)	443 (7.3%)	447 (7.1%)	567 (9.4%)	488 (8.1%)
Other service staff	721 (7.2%)	768 (6.7%)	838 (6.6%)	913 (6.6%)	1111 (8.7%)	936 (7.4%)
Other sales staff	420 (8.3%)	407 (7.4%)	462 (7.7%)	449 (7.1%)	553 (9.1%)	503 (8.3%)
Other occupation	198 (8.6%)	195 (7.9%)	212 (8.2%)	207 (7.6%)	262 (10.1%)	257 (9.9%)
Income from work in 2019						
8514-92,999 SEK	1103 (6.4%)	1016 (5.2%)	887 (3.9%)	489 (1.7%)	845 (3.4%)	1019 (4.1%)
93,000–185,999 SEK	2399 (7.9%)	2589 (7.4%)	3083 (7.2%)	2648 (5.7%)	3082 (7.1%)	2941 (6.8%)
186,000–348,749 SEK	11,289 (9.3%)	11,360 (8.6%)	12,655 (9.2%)	14,355 (10.3%)	16,654 (12.2%)	14,190 (10.4%)
348,750–464,999 SEK	5426 (7.6%)	5 447 (7.5%)	5235 (7.2%)	5606 (7.6%)	8336 (11.4%)	7233 (9.9%)
≥ 465,000 SEK	1007 (5.4%)	949 (5.1%)	903 (4.8%)	837 (4.4%)	1442 (7.7%)	1307 (7.0%)

**Fig. 1:**
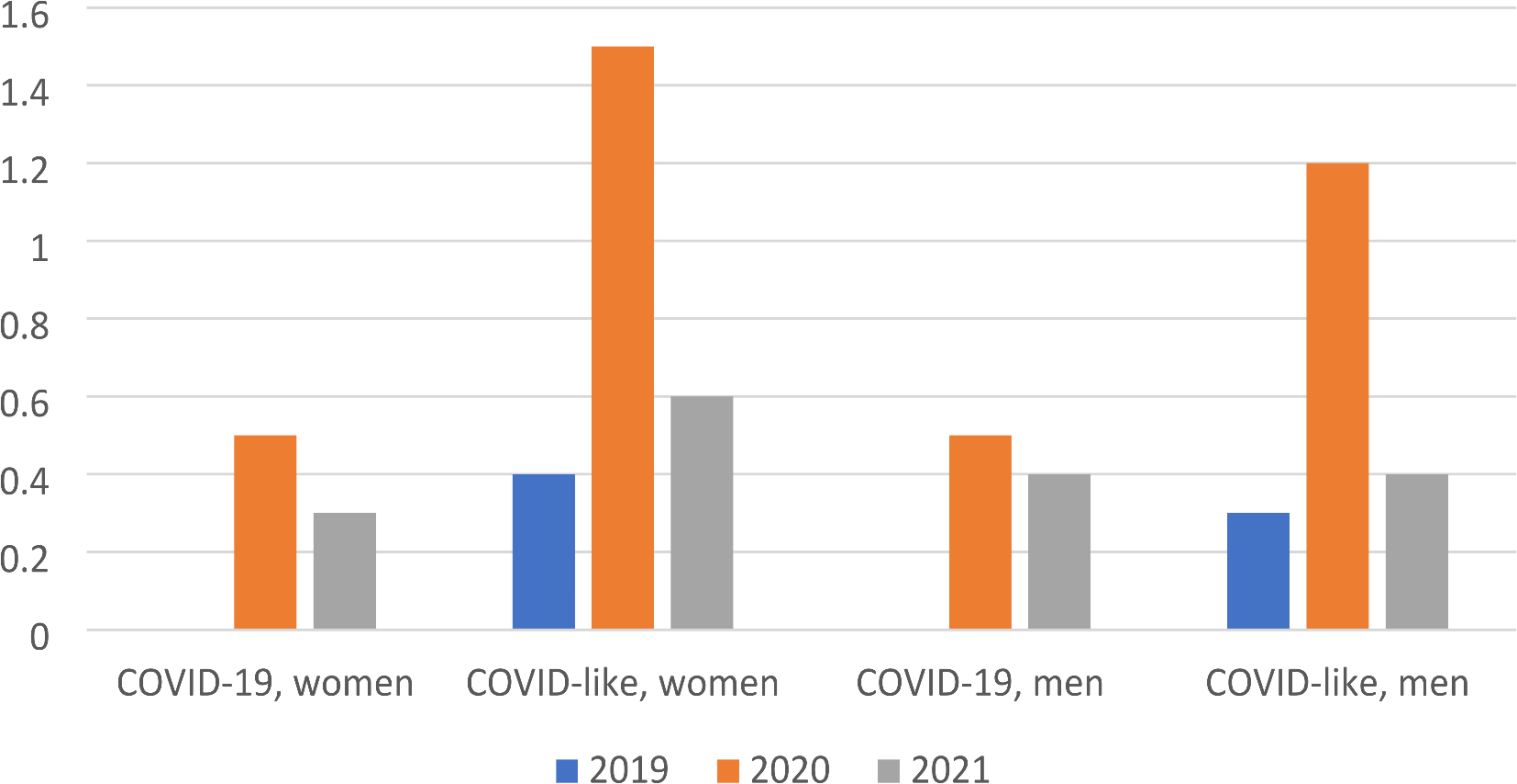
Proportion (%) of blue-collar workers in the wholesale and retail industry with sickness absence due to COVID-19 and due COVID-like diagnoses, respectively, in 2019, 2020 and 2021 among women and men.

Figure 1 shows the proportion of people with SA due to COVID-19 or COVID-like diagnoses in 2019, 2020, and 2021 among women and men. The proportion of individuals with SA due to COVID-19 was slightly lower in 2021 than 2020 among both the women and men: however, the proportion with SA due to COVID-like diagnoses other than COVID-19 was more than twice as high in 2020 than in 2019 and 2021 among women and approximately three times as high in 2020 than 2019 or 2021 among men.

Table 3 shows numbers and percent of individuals with SA due to COVID-19 or COVID-like diagnoses, as well as crude and mutually adjusted ORs for having SA due to COVID-19 or COVID-like diagnoses in 2020 and 2021 by sociodemographic factors and occupation. Men had lower odds of SA than women (adjusted OR 0.77, 95% CI 0.73–0.81), and the OR was more than twice as high among the older workers; in those aged 55–64 (2.38, 2.20–2.57) compared to those aged 25–34. Nevertheless, the oldest group 65–67 years had the second lowest odds of SA due to COVID-19 or COVID-like diagnoses, with an OR of 0.89 (0.67–1.19). Those with only high school education (1.21, 1.13–1.29) or elementary education (1.36, 1.25–1.47) had a higher likelihood of SA relative to those with a university education. Singles with children had slightly increased odds for SA (1.14, 1.02–1.27) as did those born outside EU-27 (1.47, 1.38–1.56). Those living in medium-sized towns (1.11, 1.04–1.18) or large cities (1.30, 1.22–1.39) were slightly more likely to have SA due to COVID-19 or COVID-like diagnoses than those in small towns or rural areas.

We observed few significant differences with respect to occupational categories. Warehouse and terminal staff had higher odds of SA than the reference occupation sales assistant of daily goods (1.37, 1.27–1.48), while security staff, porters, cleaners, etc. (0.74, 0.62–0.87) and other occupations (0.63, 0.47–0.84) were less likely to have SA.

All income groups were associated with elevated odds of SA compared to those with the highest income, except those with the lowest income (8514-92 999 SEK; 0.80, 0.68–0.96). The pattern was an inverse U-shape, with the highest likelihood of SA being among those in the middle-income group 186 000-348 749 SEK (1.77, 1.59–1.97).

Table 3 also shows the corresponding information for all-cause SA. In general, the associations were of similar direction and magnitudes as for SA due to COVID-19 or COVID-like diagnoses. However, some differences that were non-significant regarding SA due to COVID-19 or COVID-like diagnoses were statistically significant regarding all-cause SA: particularly regarding occupation. Warehouse and terminal staff (1.44, 1.39–1.49), transport occupations (1.32, 1.22–1.43), motor repair technicians (1.23, 1.18–1.29) and mechanics, technicians, repair, installation etc. (1.15, 1.09–1.22) all had a significantly higher risk of all-cause SA than sales assistants of daily goods.

**Table 3:** Numbers and percent as well as crude and mutually adjusted odds ratios (OR) and 95% confidence intervals (CI) of having sickness absence (SA) due to COVID-19 or due to COVID-like diagnoses and all-cause SA, respectively, during 2020 and 2021.

	SA due to COVID-19 or COVID-like diagnosis			All- cause SA		
Characteristic in 2019	n (%) with SA	Crude OR (95% CI)	Adjusted OR (95% CI)	n (%) with SA	Crude OR (95% CI)	Adjusted OR (95% CI)
Sex						
Woman	4171 (2.9%)	Ref.	Ref.	27 045 (19.0%)	Ref.	Ref.
Man	3958 (2.6%)	0.87 (0.83–0.91)	0.77 (0.73–0.81)	23 338 (15.0%)	0.75 (0.74–0.77)	0.61 (0.59–0.62)
Age						
18–24 years	852 (1.1%)	0.50 (0.46–0.54)	0.55 (0.50–0.60)	7 316 (9.3%)	0.52 (0.50–0.53)	0.57 (0.55–0.59)
25–34 years	1975 (2.2%)	Ref.	Ref.	15 179 (16.6%)	Ref.	Ref.
35–44 years	1778 (3.6%)	1.69 (1.58–1.80)	1.61 (1.50–1.72)	9 702 (19.6%)	1.22 (1.19–1.26)	1.15 (1.12–1.18)
45–54 years	1973 (4.4%)	2.10 (1.97–2.24)	2.09 (1.96–2.24)	10 116 (22.7%)	1.48 (1.44–1.52)	1.39 (1.35–1.43)
55–64 years	1501 (4.9%)	2.34 (2.19–2.51)	2.38 (2.20–2.57)	7 817 (25.6%)	1.73 (1.68–1.79)	1.62 (1.56–1.68)
65–67 years	50 (1.7%)	0.81 (0.61–1.07)	0.89 (0.67–1.19)	253 (8.8%)	0.49 (0.43–0.56)	0.50 (0.43–0.57)
Educational level						
College/university	1180 (2.2%)	Ref.	Ref.	7 236 (13.3%)	Ref.	Ref.
High school	5511 (2.7%)	1.27 (1.19–1.35)	1.21 (1.13–1.29)	34 640 (17.2%)	1.35 (1.32–1.39)	1.26 (1.22–1.30)
Elementary	1438 (3.5%)	1.63 (1.50–1.76)	1.36 (1.25–1.47)	8 507 (20.6%)	1.69 (1.63–1.75)	1.51 (1.45–1.56)
Family situation						
Single without children at home	3828 (2.1%)	Ref.	Ref.	25 519 (14.2%)	Ref.	Ref.
Single with children at home	407 (3.9%)	1.88 (1.69–2.08)	1.14 (1.02–1.27)	2727 (26.4%)	2.16 (2.06–2.26)	1.39 (1.32–1.46)
Married/cohabiting without children at home	1512 (4.3%)	2.05 (1.93–2.18)	1.03 (0.96–1.10)	8213 (23.3%)	1.83 (1.78–1.88)	1.07 (1.04–1.11)
Married/cohabiting with children at home	2382 (3.3%)	1.55 (1.47–1.63)	1.07 (1.01–1.13)	13 924 (19.1%)	1.43 (1.39–1.46)	1.04 (1.02–1.07)
Birth country						
Sweden	6319 (2.5%)	Ref.	Ref.	40 839 (16.2%)	Ref.	Ref.
Other Nordic country	115 (3.7%)	1.48 (1.23–1.79)	1.07 (0.88–1.29)	679 (21.7%)	1.43 (1.31–1.56)	1.12 (1.03–1.22)
Other EU-27	242 (3.3%)	1.32 (1.16–1.51)	1.15 (1.01–1.31)	1382 (18.8%)	1.20 (1.13–1.27)	1.08 (1.02–1.15)
Rest of the world	1453 (4.1%)	1.68 (1.58–1.78)	1.47 (1.38–1.56)	7483 (21.3%)	1.40 (1.36–1.44)	1.31 (1.27–1.35)
Type of living area						
Small town or rural	1393 (2.6%)	Ref.	Ref.	9474 (17.4%)	Ref.	Ref.
Medium-sized town	3370 (2.7%)	1.04 (0.97–1.11)	1.11 (1.04–1.18)	21 702 (17.1%)	0.98 (0.95–1.01)	1.05 (1.02–1.08)
Large city	3366 (2.9%)	1.14 (1.07–1.22)	1.30 (1.22–1.39)	19 207 (16.6%)	0.95 (0.92–0.97)	1.09 (1.06–1.12)
Occupation						
Sales assistant for daily goods	2065 (2.6%)	Ref.	Ref.	13 096 (16.8%)	Ref.	Ref.
Sales assistant for specialist trade	2464 (2.6%)	1.00 (0.94–1.06)	1.01 (0.95–1.07)	14 768 (15.8%)	0.93 (0.91–0.95)	0.95 (0.93–0.98)
Construction workers	170 (2.7%)	1.04 (0.89–1.22)	1.00 (0.85–1.18)	1109 (17.9%)	1.08 (1.01–1.16)	1.24 (1.16–1.33)
Craft workers	71 (2.2%)	0.85 (0.67–1.07)	0.75 (0.59–0.96)	545 (17.3%)	1.03 (0.94–1.14)	1.07 (0.97–1.17)
Cashiers	167 (2.8%)	1.06 (0.90–1.24)	1.08 (0.92–1.27)	981 (16.4%)	0.97 (0.91–1.04)	0.99 (0.92–1.06)
Warehouse and terminal staff	1359 (3.9%)	1.50 (1.40–1.61)	1.37 (1.27–1.48)	7472 (21.5%)	1.36 (1.32–1.40)	1.44 (1.39–1.49)
Machine and process operators	83 (2.4%)	0.89 (0.71–1.11)	0.80 (0.64–1.00)	572 (16.2%)	0.96 (0.88–1.05)	1.00 (0.91–1.10)
Mechanics, technicians, repair, installation etc.	279 (2.4%)	0.92 (0.81–1.04)	0.95 (0.84–1.09)	1837 (16.0%)	0.95 (0.90–1.00)	1.15 (1.09–1.22)
Motor vehicle mechanics and repair personnel	559 (2.5%)	0.95 (0.86–1.04)	0.98 (0.88–1.09)	3817 (17.2%)	1.03 (0.99–1.07)	1.23 (1.18–1.29)
Transport occupations	127 (2.8%)	1.05 (0.88–1.26)	0.96 (0.80–1.15)	882 (19.3%)	1.19 (1.10–1.28)	1.32 (1.22–1.43)
Security staff, porters, cleaners, etc.	151 (2.3%)	0.85 (0.72–1.01)	0.74 (0.62–0.87)	1129 (16.9%)	1.01 (0.94–1.08)	1.01 (0.94–1.08)
Other logistics	159 (2.6%)	1.00 (0.85–1.17)	0.97 (0.82–1.14)	940 (15.6%)	0.91 (0.85–0.98)	1.00 (0.93–1.07)
Other service staff	290 (2.3%)	0.86 (0.76–0.97)	1.00 (0.88–1.13)	1836 (14.4%)	0.83 (0.79–0.88)	0.97 (0.92–1.02)
Other sales staff	137 (2.2%)	0.84 (0.71–1.01)	0.94 (0.79–1.12)	938 (15.4%)	0.90 (0.84–0.97)	1.00 (0.93–1.07)
Other occupation	48 (1.8%)	0.69 (0.52–0.92)	0.63 (0.47–0.84)	461 (17.7%)	1.07 (0.96–1.18)	1.07 (0.96–1.19)
Income from work in 2019						
≥ 465,000 SEK	218 (0.9%)	Ref.	Ref.	2507 (13.4%)	Ref.	Ref.
348,750–464,999 SEK	776 (1.8%)	1.46 (1.31–1.62)	1.44 (1.30–1.61)	13 744 (18.8%)	1.50 (1.43–1.57)	1.41 (1.35–1.48)
186,000–348,749 SEK	4431 (3.2%)	1.50 (1.36–1.67)	1.77 (1.59–1.97)	26 990 (19.7%)	1.59 (1.52–1.66)	1.65 (1.57–1.72)
93,000–185,999 SEK	2296 (3.1%)	0.81 (0.72–0.92)	1.36 (1.20–1.55)	5410 (12.4%)	0.92 (0.87–0.96)	1.23 (1.17–1.30)
8514-92,999 SEK	408 (2.2%)	0.39 (0.33–0.46)	0.80 (0.68–0.96)	1732 (6.9%)	0.48 (0.45–0.51)	0.75 (0.70–0.80)

## Discussion

In this explorative longitudinal cohort study of sickness absence (SA) among all blue-collar workers in the retail and wholesale industry before and during the COVID-19 pandemic, we found that their SA rates increased slightly in 2020 and 2021, relative to previous years. Women, older individuals, those with lower education, those born outside EU-27, those with lower or middling income, and warehouse and terminal staff had higher odds of having both SA due all diagnoses and specifically due to COVID-19 or COVID-like diagnoses, after adjusting for numerous factors. Moreover, those living in larger cities had a higher odds of SA due to COVID-19 or COVID-like diagnoses.

Even though the proportion of individuals with SA was elevated in 2021 compared to 2016–2019, it was lower than in 2020. This was especially the case for SA with COVID-like diagnoses excluding COVID-19, indicating that the diagnosis of COVID-19 had become more reliable. However, rates of SA due to diagnosed COVID-19 were also lower in 2021 than in 2020, even though the rates of people being diagnosed with COVID-19 were still high^32^. Vaccines for COVID-19 were approved for use in late 2020 and were rolled out to the public throughout the spring of 2021^33^. The vaccines were successful in slowing the spread of COVID-19, and importantly in preventing serious symptoms from developing when infected^34^. Furthermore, the viral mutation that came to dominate the spread of infection, known as omicron, was generally associated with a milder version of the disease than previous variants of the virus^35^. These two factors might have influenced our results for 2021, if those who had COVID-19 had less severe symptoms and therefore fewer needed SA > 14 days. The limited rates of testing in the beginning of the pandemic and the development of treatments for severe COVID-19 during the course of the pandemic make comparisons between the years less reliable, however, discoveries of treatment methods and vaccines might have led to shorter durations of sickness^36^ and, therefore, potentially also of SA due to COVID-19.

A study of individuals aged 18–64 in Sweden with a positive polymerase chain reaction (PCR) test of COVID-19 found that only 5.7% of them had SA > 14 days within 7 days before and 30 days after their test^6^. This means that the vast majority of individuals with COVID-19 either were able to carry out their work tasks with a COVID-19 infection, possibly by working from home, or that they were able to return to work within 14 days, and thus did not receive SA benefits from the Social Insurance Agency. Similarly, a study from Germany found that only 6.5% of women and 5.1% of men diagnosed with COVID-19 were on SA for more than 4 weeks^1^. Data from the Swedish Social Insurance Agency indicates that, for the SA spells they had information on, SA spells due to COVID-19 were generally shorter than those due to other diagnoses^5,30,37^, meaning that it is highly plausible that most SA spells due to COVID-19 were ended within 14 days, and were thus not included in this study. This means that SA (especially > 14 days) cannot be used as a measure of infection rates or prevalence of disease: it is a measure of reduced work capacity due to that disease. The spells that lasted > 14 days and could be included in this study were thus most likely the more severe spells of COVID-19 and COVID-like diagnoses, given the rarity of SA > 14 days among those with confirmed COVID-19.

In general, the associations between sociodemographic factors and SA due to COVID-like diagnoses were very similar as those found for all-cause SA. They were also in the same direction as those found in other studies examining SA due to other diagnoses^24,26,27^. For example, women were more likely to have SA than men, and there were educational and age-related gradients (people with lower education and in higher ages were more likely to have SA). Other studies have also found that the occupational and socioeconomic patterns of SA with COVID-19 mirror those of SA in other diagnoses before the pandemic^9,10,37^. The one exception to this was living in cities, which was associated with a higher OR of SA in our study, both for all-cause SA and SA due to COVID-19 or COVID-like diagnoses (although only very weakly for all-cause SA) but which previously has been implicated with lower likelihoods of SA when compared to rural areas^24,26^, including in a study of white-collar workers in the retail and wholesale industry^27^. Similar results of regions with high population density having higher rates of SA due to COVID-19 were also found in France^2^. The role of population density in the transmission rates of COVID-19 has been disputed: while it might increase contact frequencies between people, high infection rates and dense areas might mean people isolate more strongly^38^. A study from the US concluded that population density contributed to faster transmission in the first months of local outbreaks, but not after^38^. With regards to Sweden, Stockholm was hit hard and early in the pandemic, due to interconnectivity and international travel to and from Stockholm, especially before travel restrictions and public recommendations were implemented^39^. This may have contributed to the higher rates of SA in large cities in this study. Public transport is one of the sources of virus transmission, and can thus contribute to more widespread transmission in cities, where public transport networks are denser^38^. The use of public transport in Stockholm fell dramatically during 2020 compared to the same months in 2019, although less so in lower socio-economic areas^40^. However, many of the larger shopping centres are concentrated in the larger cities in Sweden and the risk of contact with other people with COVID-19 would have been higher, especially among those working with contacts with customers or the public.

That women had higher rates of SA due to these diagnoses than men reflects the pre-pandemic situation in general as well as in several occupations^24,26,27,41^ and is in line with findings in other studies on SA during the pandemic^1,3,42^. However, a study of SA in Sweden during the early part of the pandemic (March-August 2020), found that a higher proportion of those with more than 12 weeks of SA were men, although that study only considered SA due to COVID-19 (not including the diagnoses that were also used for suspected COVID-19 before the advent of mass testing)^3^. On the other hand, another study found that women had more recurrent and longer SA due to COVID-19^43^, and yet another study found that women had more persistent fatigue after a COVID-19 infection^1^. Finally, a study of healthcare and residential staff in Sweden found that the association between sex and SA > 3 weeks was non-significant^44^. Men had a higher risk of more severe COVID-19 in terms of hospitalisation^1^ and mortality, especially among people below 60 years of age^45^, however, the gender differences regarding SA are not as clear, and need to be studied in more detail.

Previous research has identified that individuals in occupations with many contacts with other people had a higher risk of SA due to COVID-19 – although this was particularly true among people working in healthcare and elder care, it was also the case for cashiers and others working in the sale of daily goods^9,10^. However, we found that warehouse and terminal staff had a significantly higher risk of SA due to COVID-19 and COVID-like diagnoses than sales assistants of daily goods, while there were very few other significant differences based on occupation. A study from the USA found that workers in the transportation and warehousing sector were among those with the highest risk of filing for Worker’s Compensation claims for work-related COVID-19 infection^46^. The same study also found that occupations with close proximity between workers and high physical activity (which would likely involve heavy breathing) were associated with clusters of several outbreaks of COVID-19 at the same workplace^46^, both of which may apply to warehouse workers in our study population and contribute to the elevated ORs of SA > 14 days due to COVID-19 or COVID-like diagnoses that we observed. Warehouse and terminal staff also had higher rates of all-cause SA than all other studied occupational groups, both before and during the pandemic. This is in line with what other studies have found regarding the situation prior to the COVID-19 pandemic, where individuals working in transport and logistics occupations had higher rates of SA spells > 90 days due to respiratory diseases than individuals working in commerce occupations^47^. Similarly, another study from Sweden found that packers, loaders and warehouse workers had higher risk of specialist healthcare due to asthma than many other occupational groups^48^. This indicates that they might have a higher risk of respiratory conditions in general, which may have also impacted their risk of SA due to COVID-19 or COVID-like diagnoses during the pandemic in our study. We also found that warehouse and terminal staff also had elevated ORs of all-cause SA in our study, which adds support to this potential explanation.

We found only modest changes in the odds ratios between the crude and fully adjusted models, indicating that the associations between occupation and all-cause SA and SA due to COVID-19 or COVID-like diagnoses, respectively, are not much affected by educational level or other sociodemographic variables.

That income level is related to SA has been established in several previous studies, and most of these studies show a gradient whereby people with lower income levels have higher risk of SA^49–52^. The reasons for this are many, including material deprivation, exposure to psychosocial stress, and differences in health-related behaviours. For example, those with higher incomes tend to both have better health, and be more able to deal with the consequences of disease and injury^53,54^. In our study, we found that nearly all income groups had a higher risk of SA than the highest income group, which is in line with this explanation, although we did not find a strong gradient. On the other hand, those with the lowest incomes had the lowest risk of SA due to COVID-19, which is contrary both to previous findings about the social gradient in SA^55^, and the social gradient of COVID-19^56^. This could be because they worked part-time or only part of the year, and were thus to a lesser extent at risk of needing to be on SA due to COVID-19 or other diagnoses, something we cannot explore with our data. This is also related to the very low income level we used for inclusion, as well as the skewed income distribution in our study, with many in the low-income groups and fewer in the high-income groups.

The results from this study show that overall SA rates increased slightly during the pandemic, and that the increase in the proportion of individuals with all-cause SA corresponded to the proportion of individuals with SA due to COVID-19 or COVID-like diagnoses. However, the sociodemographic patterns in SA during the pandemic were largely similar to those before, and there may of course have been other changes that occurred during 2020–2021, unrelated to the pandemic, that influenced the SA rates^5,57^. The results add to the findings from other studies on other outcomes, showing that the sociodemographic and socioeconomic patterning of COVID-19 and the consequences of COVID-19 were in line with previously observed health inequalities^58^, indicating that it is important to study different health outcomes and the social patterning of them when new diseases such as COVID-19 emerge, and especially to pay attention to those groups with already high risks of outcomes such as disease, poor health, and, as this study shows, sickness absence.

## Strengths and limitations

The main strength of this study is that it draws from a fully enumerated population-based cohort including all 297 378 individuals in Sweden eligible for inclusion. This means that the study is not based on a sample, and that the study population was large enough for subgroup analyses. Furthermore, all individuals could be followed up from inclusion until they died, emigrated, or to the end of follow-up (i.e., no drop-outs). Other important strengths are that we could use linked microdata from three nationwide administrative registers of good quality^25,59^, and that no self-reported data were used, thus eliminating the possibility of recall bias.

The study also has some limitations. In this longitudinal cohort study, we included those who were employed in blue-collar occupations in retail and wholesale in 2019: this means that for the analyses of other years (2016–2018 and 2020–2021), we may have missed individuals who fulfilled the inclusion criteria during that year, but not in 2019. Furthermore, we only had information on income on an annual basis. Therefore, if individuals were eligible for part of the year (e.g., students who worked during the summer months, or older individuals who retired to old-age pension partway through the year), they were included as eligible during that year, and their SA was measured on an annual basis. This means that some of those who were included in the population that was eligible during a year, were not eligible the entire year.

That we could not include SA spells shorter than 15 days can also be seen as a limitation, especially since many SA spells due to COVID-19 were relatively short compared to those of other diagnoses^5,30,37^. However, it can also be seen as a strength that we only include the longer, more severe SA spells that in many cases have been physician-certified. One limitation is the reliability of the COVID-19 diagnosis: during the early stages of the pandemic, before the introduction of mass testing, many individuals who had suspected COVID-19 were given other SA diagnoses on the medical certificate. We therefore followed the practice of the Social Insurance Agency and included some infectious, respiratory, and symptom-based diagnoses as COVID-like diagnoses to also capture these individuals; however, this may have resulted in misclassification and an overestimation of COVID-like SA. On the other hand, there may have been other diagnoses used than those the Social Insurance Agency classified as COVID-related, which would then have led to an underestimation of COVID-like SA. Since COVID-19 affects many organs and expresses itself through many symptoms^60^, there are possibly other potential diagnoses that could be used for symptoms related to COVID-19 when COVID-19 status is not confirmed.

We have only investigated all-cause SA and SA due to COVID-19 and COVID-like diagnoses in this study. It is quite possible that there were changes to SA rates in other diagnoses that we were unable to distinguish by grouping most SA diagnoses together. Such possible changes to diagnosis-specific SA rates could be either related to or unrelated to the COVID-19 pandemic and the response to the pandemic. Future research should investigate other SA diagnoses in more depth, and also include other factors unrelated to the COVID-19 pandemic that may influence SA rates on a societal level, such as the business cycle, social security and labour market regulations, etc.

Since the study population consisted of privately employed blue-collar workers in the retail and wholesale industry in Sweden, the results cannot directly be generalized to other types of occupational populations or to countries with other SA systems or employment frequencies.

## Conclusion

In this investigation of SA among blue-collar workers in the wholesale and retail industry before and during the COVID-19 pandemic, we found that SA due to all diagnoses increased marginally during 2020 and 2021 relative to the four prior years. Patterns of SA by sociodemographic factors and occupation remained similar to previous years. Warehouse and terminal staff had the highest risk for SA due to COVID-like diagnoses. Given that this group also had higher rates of SA prior to the pandemic, this could be a group that warrants further investigation.

## Data Availability

The used data cannot be made publicly available due to privacy regulations. According to the General Data Protection Regulation, the Swedish law SFS 2018:218, the Swedish Data Protection Act, the Swedish Ethical Review Act, and the Public Access to Information and Secrecy Act, these types of sensitive data can only be made available for specific purposes that meet the criteria for access to this type of sensitive and confidential data as determined by a legal review. Contact imas-cns@ki.se for information regarding the data.
